# Book Reviews

**Published:** 1812-07

**Authors:** 


### CRITICAL ANALYSIS

### *OF RECENT PUBLICATIONS*

IN THE

### DIFFERENT BRANCHES OF PHYSIC, SURGERY, AND MEDICAL PHILOSOPHY.

*Medico-Chirurgical Transactions, published by the Medical and Chirurgical Society of London.* Vol. 2. 8vo. pp. 420. plates. Longman and Co. 1811.

THE short interval which has intervened since the publication of the first volume of these Transactions, indicates the activity, as the variety and quality of the communications denote the talent, of the members of the Medical and Chirurgical Society. By this time the volume has doubtless been perused by the reading part of the profession in London: for the benefit of those whose situations in the country preclude them from ready access to books, we shall enrich our pages with some extracts from the articles which, in our opinion, possess the greatest claim to notice.

The volume opens with a case of aneurism by anastomosis in the orbit, by Mr. Travers.

"Frances

"Frances Stoffell, aged 34, a healthy active woman, of fair complexion, middle stature, and the mother of five children, on the evening of the 28th of December, 1804, being some months advanced in pregnancy, felt a sudden snap on the left side of her forehead, which was attended with pain, and followed by a copious effusion of a limpid fluid into the cellular substance of the eyelids on the same side. For some days preceding she had complained of a severe pain in the head, which was now increased to so great a degree, that for the space of a week she was unable to raise it from the pillow. The œdematous swelling surrounding the orbit was reduced by punctures; an issue was set in the temple for a smart attack of ophthalmia which supervened; and leeches and cold washes were applied. She now first perceived a protrusion of the globe of the eye which affected the sight, and a circumscribed tumor, elastic to the touch, about as large as a hazel nut, appeared upon the infra-orbitary ridge. Another softer and more diffused swelling arose, at the same time, above the tendon of the orbicularis palpebrarum. The lower tumor communicated both to the sight and the touch, the pulse of the larger arteries; the upper gave the sensation of a strong vibratory thrill. The swellings grew slowly, and the skin between the eyes and that of the lower eyelid became puffed and thickened. The globe of the eye was gradually forced upwards and outwards, and its motions were considerably impeded. She had a constant noise in her head, which, to her sensation, exactly resembled the blowing of a pair of bellows. The pulsatory motion of the tumors was much increased by agitation of mind, or strong exercise of body. But the most distressing of her symptoms was a cold obtuse pain in the crown of the head, occasionally shooting across the forehead and temples. She was compelled to rest the left side of her head on her hand when in the recumbent posture, and found the beating and noise to increase sensibly when her head was low and unsupported.

"Her physiognomy was hard and coarse, and the skin in the region of the orbits appeared morbidly thick and wrinkled. The eye-brow of the diseased side was straitened, and driven from two or three lines above the level of the opposite eye-brow. The upper half of the inner canthus was filled by the thrilling tumor, which presented a loose woolly feel, was very compressible, and when firmly compressed offered a slight pulsation. The veins of the superior lid were varicose from distention; the skin was much pursed over the lacrymal sac; and the veins on the side of the nose turgid. The lower tumor, which projected above the suborbitar hole, was of a conical shape, and firmly elastic to the touch. The under lid was raised as far as to the outer angle of the orbit, above the apex of the cheek. This lower tumor could be emptied or pressed back into the orbit, but the pulsation then became violent; and, from the increased pressure of the globe upon the roof and side of the orbit, the pain was insupportable. Careful compression of the temporal, angular, and maxillary, arteries, produced no effect on the aneurism."

Upon applying his thumb to the trunk of the common carotid, Mr. Travers found the pulsation ceased. Persuaded that the disease was similar to that described by Mr. John Bell under the term "Aneurism by Anastomosis," and finding no good effect result from either pressure, or cooling applications, Mr. Travers determined to place a ligature on the carotid artery.

"The patient was laid supine, the neck raised by a pillow, the chin slightly turned to the left shoulder. An incision, about two inches and a half in length, was commenced at the distance of one inch above the external extremity of the clavicle, and carried in an oblique direction along the anterior edge of the mastoid muscle. The fibres of the muscle being exposed, its edge was raised, and the sheath of the vessels cautiously cut open on the tracheal side. Through this opening, which was of very small extent, a curved eyed probe, carrying a stout round ligature, was passed beneath the artery,* care being taken to exclude the nerve. The probe being cut away, the ligatures were drawn apart from each other, the lower being tied at the lowermost point of the denudation of the artery, the upper at the highest. They were about one fourth of an inch distant, and whilst they were tightened, the division of the internal coat of the vessel could be distinctly felt. The lips of the wound were lightly brought together by adhesive straps, and the ligatures drawn out opposite to the point of their application on the artery."

This important operation completely succeeded. An engraving furnishes a tolerable representation of the disease previous to the operation, and the second figure shews the nearly perfect state of the eye two years afterwards.

A fatal case of Hydrocephalus Internus, by Mr. Cooke, of Brentford, combined with a scrofulous affection of the liver and left kidney, was curious from the circumstance of the patient, a girl six years of age, having so early as her fourth year the external pudenda prominent, and covered with a quantity of dark hair. Her upper lip was also well furnished with hair, her voice was strong, and her whole appearance, except the breasts and general stature, was that of puberty.

Dr. Wall, of Oxford, has also added to the catalogue of extraordinary cases, by communicating an account of an infant menstruating at nine months, and in her second year possessing all the external signs, except of expression and stature, which characterise woman.

The success of large doses of oil of turpentine in tœnia

---

* The pulsation of the lower tumor immediately ceased upon compressing the vessel with the finger as it lay over the probe.

is is rendered indisputable by several authenticated cases which came under the notice of Dr. Fenwick, of Durham.

Dr. Bateman has related an instance of secondary small-pox, and has given several references to authors who have recorded similar facts.

A case of ununited fracture of the thigh, cured by sawing off the ends of the bone, is communicated by Mr. Griffith Rowlands, of Chester.

Mr. Burrow has stated the particulars of a curious case of of what he terms "Hernia cerebri."

"The head of the child was remarkably deformed; the whole of the forehead, summit, and a great part of the occiput, were deficient, and, in lieu of them, a substance projected of a light mulberry color, and of the mushroom form, excepting that its neck was proportionably broader. From the deficiency of bone, the eyes appeared to project much more than usual; the body of the child had its usual color, and in every other respect it was naturally formed.

"The child lived six days without either taking sustenance or having any evacuation. Attempts were frequently made to give it food; but when the smallest quantity entered its throat, it excited convulsions and immediate regurgitation."

Respiration went on naturally, and the pulse did not differ from that of other infants. Whenever the projection on the head was touched, a general and violent convulsion was produced.

Dissection presented the following appearances:

"The scalp, the os frontis, the parietal, and a great part of the occipital, bones were wanting. Through the parts at which these bones were deficient, the cerebrum projected; and this portion of the brain exhibited the usual convolutions of that substance. It was covered by the pia mater; it was of a mulberry color; appeared to be more vascular than the pia mater usually is; and the edge of the scalp was united with the neck of the tumor.

The cerebellum was not more than one fourth of its usual size, for the posterior part of the os occipitis had advanced towards the sella turcica, so as to form a cavity for the cerebellum, into a canal about twice the size of that which is destined for the spinal marrow."

The child was deprived of the power of voluntary motion, and the secretions were entirely stopped.

In a case of wound of the heart, by Mr. Heatherton, it appears that a soldier in falling was pierced by a bayonet between the sixth and seventh ribs. Mr. H. saw him five minutes after the accident in a state of syncope, with cold extremities, and a pulse scarcely perceptible. He revived in about a quarter of an hour, did not complain of any severe pain, and expressed "that he believed he was more frightened than hurt." Mr. H. on examining the wound, could not trace its extent farther than one inch and a quarter, though it was evident the bayonet had penetrated two inches. The hæmorrhage was inconsiderable. He could not lie on his right side, but slept well.

"On visiting him the following morning, he complained of lancinating pains extending from the wounded part across the chest, and of severe fugitive pains in different parts of the abdomen, his pulse was quick and thready, and tongue white and dry: these symptoms led to a suspicion that the pleura costalis, at least, was wounded, though no opening could be ascertained extending into the cavity of the chest. Sixteen ounces of blood were taken from his arm, a solution of magnesia vitriolata administered, and fomentations applied to the abdomen. He was obliged to be supported in bed nearly in a sitting posture, as respiration became much impeded when perfectly horizontal: in this position he appeared to breathe with freedom." In the evening he felt much relieved, passed a good night, and continued throughout the following day walking about and conversing in high spirits. "He retired to rest about nine o'clock, and fell asleep; at eleven he got out of bed to the commode, had an evacuation by no means costive, said 'he felt himself chilly, and a sensation that he should die,' returned into bed, and expired immediately; making a period of forty-nine hours, from his first receiving the wound."

*Dissection.*—"On opening the chest, the pleura was found slightly inflamed for some distance around the puncture, and an effusion of coagulable lymph, uniting a small portion of the lung to the wounded part: the lung, however, was uninjured. At least two quarts of blood were effused into the cavity of the chest; the pericardium was nearly filled with blood, and had a puncture through it, extending three quarters of an inch into the muscular substance of the left ventricle, about two inches from its apex. A small coagulum of blood was formed at the edge of the wound through the pericardium. Upon opening the left ventricle of the heart, the bayonet was found to have penetrated the substance of the ventricle, and to have cut one of the fleshy columns of the mitral valve."

A case of extraordinary enlargement of the right lower extremity, by Mr. Chevalier, is related with great correctness, and is curious from its uncommonness. The bulk chiefly arose from a growth of skin and adipose membrane.

Mr. Chevalier states, that, since this case occurred, he has seen an instance of the true Egyptian Elephantiasis, according in every particular with the description given by Mr. Bruce, in his Travels. On examining the skin after death, Mr. C. found that

"The change produced was chiefly in the papillæ of the cutis, each of which appeared elongated and enlarged into a roundish tubercle, over the surface of which a thick and almost horny cuticle grew, grew, giving to the surface its dark colour and rough appearance. In the upper part of the leg, these tuberculated papillæ were smaller, and the cuticle thinner; but in the lower part they were large, and the tumefaction was so great, that the foot projected very little beyond the leg, more than the length of the toes. These larger tubercles were exquisitely painful in themselves, and were undoubtedly rendered more so by the horny cuticle dipping down into all their interstices and pressing upon them.

"From this account, it will appear that the true Elephantiasis is a disease totally distinct from some cutaneous affections which have been called by that name. One of them is an excoriated state of the skin, which forms an imperfect cuticle, or rather a hard crust, not much unlike, in its appearance, to the true Elephantiasis; but, when this crust is separated, the cutis underneath is found smooth, and but little, if at all, changed in its structure. This occurs mostly in the leg, but sometimes takes place in other parts."

These cases are illustrated by appropriate engravings. Mr. Chevalier has also stated a case of Lithotomy, with some judicious remarks on the effect of the operation; and on some cases of Fistulæ in perineo.

Dr. Marcet has related a severe case of Erythema, unconnected with mercurial action. From which he is induced to think, "that the names Erythema mercuriale, or Hydrargyria, which have been applied to this disease, are to be considered as expressing a variety rather than a species of disease." We do not object to this opinion; but Dr. Marcet does not seem to be aware that it is already well known that the complaint in question, may in certain habits be produced when a very small quantity of mercury has been taken; and, from his own account of the case, we infer that his patient had taken mercury in some form or other. Thus the doctor states, "when he (the patient) was attacked for the first time, he was just recovering from a gonorrhœa, for which he had used some internal remedies, none of which had affected his mouth, or produced any salivation." Now, Mr. Pearson has remarked, that he has seen the disease occur partially from touching any part of the body with mercurial ointment, and even by a few grains of red precipitate falling accidentally on the skin. A case is recorded by Bonetus, in which the disease followed the application of mercurial ointment to the head to destroy pediculi; and Dr. Alley, in his recent publication, has satisfactorily established the existence of a peculiar idiosyncracy disposing to the affection on the application of very small doses of mercury, either externally or internally; and has also described very similar affections produced by causes wholly independent of mercurial action.

Dr.

Dr. Marcet has communicated the history of a very singular nervous affection which occurred in the person of Dr. Vieusseux, of Geneva, who drew up the particulars of his own case, which, however, is too long to state, and cannot be abridged with advantage.

We are also indebted to Dr. Marcet for a chemical account of various dropsical fluids, with remarks on the nature of the alkaline matter contained in these fluids, and on the serum of the blood. The experiments appear to have been accurately conducted, and we have great satisfaction in presenting our readers with the author's general view of the whole.

"It appears, in the first place, that the prevailing animal substance, not only in serum, but in all the morbid fluids which have been examined in this essay, is albumen, or coagulable matter; which substance, however, these fluids contain in very different proportions.

"In all of them, also, another kind of animal substance, which may be called muco-extractive matter, (from its being incoagulable, and from its being soluble in water or other menstrua,) is uniformly discoverable.

"Gelatine, it would appear, is not discoverable in any of these fluids; a singular circumstance; from which it seems natural to infer that the formation of gelatine is the result of a specific secretion.

"In some of these fluids, namely, those of ascites, hydrothorax, hydrops pericardii, hydrocele, and that which is sometimes effused in the thyroid gland, the albuminous matter is so considerable as to render them coagulable; that is, convertible into an uniform semi-solid mass, by the agency of acids, or by a temperature of 165 degrees. In others, on the contrary, namely, in the fluid of spina bifida, of hydrocephalus, and of hydatids, the quantity of albuminous matter is so small, as scarcely to be rendered visible by heat or acids."

Dr. Marcet also proved that the specific gravity of these fluids is extremely various; and that the differences principally affect the animal matter of these fluids; the saline matter not being subject to similar variations.

"The particular saline ingredients contained in all these fluids, appear to be, muriat of soda, muriat of potash, sulphat of potash, soda, and phosphats of lime, iron, and magnesia. And a mass of 100 grs. of these salts appears to consist of about 72 grs. of muriat of soda, mixed with a little muriat of potash, between 18 and 20 grs. of soda, brought to the state of subcarbonat; and a mixture of 8 or 10 grs. of sulphat of potash, phosphat of lime, phosphat of iron, and phosphat of magnesia. Potash, therefore, as Dr. Pearson first stated, is present in the animal fluids; but I believe I have satisfactorily shewn, that it exists in the state of muriat, or sulphat; soda being the only alkali discoverable in an uncombined state."

The subjoined Table shews the proportions of saline and animal matter in various dropsical fluids, and in the serum of the blood.

| | In 1000 grs. of Fluid. | | | |
|---|---|---|---|---|
| | Specific Gravity. | Total of Solid Contents | Quantity of Animal Matter. | Quantity of Saline Matter. |
| | | Grains. | Grains. | Grains. |
| Fluid of Spina Bifida . . . | 1007 | 11,4 | 2,2 | 9,2 |
| ——— Hydrocephalus . . . | 1006,7 | 9,2 | 1,12 | 8,08 |
| ——— Ascites . . . . . . | 1015 | 33,5 | 25,1 | 8,4 |
| ——— Hydrothorax . . . | 1012,1 | 26,6 | 18,8 | 7,8 |
| ——— Hydrops Pericardii . | 1014,3 | 33 | 25,5 | 7,5 |
| ——— Hydrocele . . . . | 1024,3 | 80 | 71,5 | 8,5 |
| Serum of Blood* . . . . . | 1029,5 | 100 | 90,8 | 9,2 |

Dr. Bree has contributed some useful practical remarks on painful affections of the side from tumid spleen, with a case explanatory of the remote cause of the affection, and the manner of treating it with success.

Mr. Bush has communicated the case of a sailor, in the muscles of whose back the blade of a knife lodged above thirty years, producing so little inconvenience, that he was enabled to perform the duties of a seaman several years, and afterwards follow the trade of a weaver.

A case of fracture of the occipital bone, extending to the great foramen; in which that bone was trephined, and the dura mater of the cerebellum punctured, stated by Dr. Hutchinson, surgeon to the Royal Naval Hospital at Deal, will be perused with much interest. The operation completely succeeded, although Mr. John Bell has observed that he *always found it fatal.*

Dr. Henry, of Manchester, has contributed a valuable paper, containing the report of various experiments which he had instituted, with the view of determining more precisely the contents of the urine voided in diabetes. Much as this subject has been discussed of late, we are still very ignorant of the real nature of the disorder, and very undetermined respecting the best mode of treating it.

Dr. Roget has stated the particulars of a very interesting case, the subject of which, a young woman, had swallowed sixty grains of white arsenic, in powder, sprinkled on bread and butter. In ten minutes vomiting was produced; in about

* The specific gravity here stated is an average. That of the particular specimen under examination was 1024,5.

an an hour this increased in severity, and was attended with griping in the bowels, and copious watery evacuations by stool. The patient was in great agony, and took, by the direction of an apothecary, five grains of sulphur in a pill, together with three spoonfuls of a mixture of ℥ſs. of sulphat of magnesia, ℥j. of subcarbonat of potass, and a grain of tartarised antimony, in ℥viij. of peppermint-water. Three doses of this mixture were taken in the course of the night.

Dr. Roget saw her about twelve o'clock on the following day, and found her "suffering intense pain at the pit of the stomach, much increased by pressure, and accompanied with frequent retching, and occasional fits of vomiting. There was general tension over the abdomen. The face was flushed, the respiration hurried, anxious, and often interrupted by spasmodic catchings approaching to hiccup. The pulse was 120, small, and extremely quick; the tongue white, but moist. The voice was tolerably firm, and the speech perfectly distinct and collected." A vein was opened in the arm; when eighteen ounces of blood had been drawn, the patient grew pale, complained of being sick, and vomited about half a pint of fluid of the appearance of thin gruel. She fainted, and remained about half an hour in a state of insensibility. Upon recovering from the syncope, she had less pain of the stomach, and the sickness was entirely removed. She complained much of chilliness, especially in the extremities, although to another person they felt of the natural temperature.

A large blister was applied over the region of the stomach. In the evening the pain in that viscus, though occasionally remitting, was intense, with slight efforts to vomit. There was also pain in the fore part of the head; deglutition was painful from a burning sensation in the fauces, which extended to the chest and stomach; the mouth was clammy, and great thirst was felt, though the saliva was plentiful. The skin was hot, pulse 120, and wiry. Light occasioned great uneasiness. Being costive, ℥j. of Ol. Ricini was given in divided doses.

She continued for several days suffering urgent pain, with occasional remissions; extreme lowness, syncope, and convulsive fits: for the detail of which, and the successful termination, we refer to Dr. Roget's very methodical Report, and shall now confine ourselves to the statement of his ingenious reasoning upon the case, which is memorable from the large quantity of arsenic that was swallowed.

"We may remark (observes Dr. R.) that the first operation of the arsenic was that of a violent emetic and purgative; and to this circumstance I am inclined to think the patient owed her only chance chance of safety. The corresponding effects began to take place about an hour after its reception into the stomach, and were promoted by copious dilution, by which I should imagine that nearly the whole of the poison was evacuated. It appears, indeed, from the testimony of a number of authors, that a few grains is in general sufficient to occasion death: had any considerable portion of the sixty grains, therefore, remained longer upon the stomach or intestines, the consequences would have been irretrievably fatal. From the analysis of the fluid vomited at the time I first saw the patient, and of which I shall afterwards give an account, it would appear that none of the arsenic was at that time present in the stomach.

"But the impression which the poison had made upon the coats of the stomach, was not of a nature to subside on the removal of the cause that had produced it. The inflammation excited in that organ, and the symptoms of which, at the time I first saw the patient, were strongly marked, would probably, if left to its natural course, have run through all its stages, and terminated in gangrene. I considered that dilution had been largely employed, and that evacuations from the stomach and intestines had been abundant. The stomach was in so irritable a state, that no medicine could be exhibited with any prospect of advantage. I concluded that the principal and more immediate source of danger consisted in the inflammation of the stomach; and, viewing the matter in this light, was induced to adopt the treatment proper for idiopathic gastritis. The bleeding was pushed ad deliquium; a large blister was applied to the epigastric region, and the bowels kept open by frequent doses of Oleum Ricini. The employment of these remedies was productive, indeed, of great debility, which, however, was but temporary; the advantages procured were more permanent; these were the occasional remission of the pain, and the immediate cessation of the vomiting, which never afterwards returned, excepting once in the course of the following night. An effectual check was given to the inflammatory process, and a remission of the symptoms was obtained for about thirty-six hours; during which the system shewed a tendency to return to its natural condition.

"To this state of comparative convalescence, there succeeded a new train of symptoms, threatening danger of a different kind, but of no less magnitude than the former. These were of a nervous nature, apparently resulting from the sedative action of the poison upon the nervous system. To this cause may be referred the sense of coldness and numbness over the body, of void in the stomach; the somnolency, the severe head-ach, the tremors and convulsions; the anxiety and constant tendency to fainting. These, conjoined with the deadly paleness of the countenance, inspired me with apprehensions that Nature would sink under the struggle. Assistance might have been afforded by stimulant remedies; but these were contra-indicated by the presence of many symptoms of irritation in the throat and primæ viæ, by the sensation of burning heat in these parts, by the hardness of the pulse, and occasional sharp pains in the region of the stomach, which implied that a disposition to inflammation still existed. I therefore ventured only upon small doses of camphor with peppermint water; and from these medicines relief was evidently experienced.

"In the course of a day or two the above-mentioned symptoms had subsided; but it was only to give place to another state of disease of as formidable a kind. The pain of the stomach had gradually extended to the lungs, and was now accompanied with all the characters of pneumonic inflammation. Notwithstanding the exhausted condition of the patient, I had again recourse to the antiphlogistic treatment, conjoined with diaphoretics, and with the same success as before. The pneumonia was subdued, and weakness was all that remained.

"It might now have been presumed that no further obstacle to recovery would arise, and that the arsenic had exhausted its power of doing mischief. The fallacy of such expectations was soon apparent. It was still lurking in the system, although some days elapsed without its exhibiting any marked effect. On a sudden its activity was again exerted, and a new order of symptoms of the convulsive kind were excited. The fits were completely epileptic, and were accompanied and followed by insensibility, which in the beginning was of such long duration as to excite fears for the event. These apprehensions were dissipated by the fits becoming every day shorter, and the recovery from each more rapid and complete. In the course of a week they had entirely ceased, and the patient has since remained free from any urgent complaint; although the constitution has evidently been injured by the deleterious influence of the poison."

Dr. Roget concludes his paper with some observations upon the tests of arsenic, and describes one which was suggested by Dr. Marcet in the present case, as being of peculiar delicacy. The directions for using it are as follow:

"Let the fluid, suspected to contain arsenic, be filtered; let the end of a glass rod, wetted with a solution of pure ammonia, be brought into contact with this fluid; and let a clean rod, similarly wetted with a solution of nitrate of silver, be brought into contact with the mixture. If the minutest quantity of arsenic be present, a precipitate of a bright yellow color, inclining to orange, will appear at the point of contact, and will readily subside to the bottom of the vessel."

The yellow appearance was distinctly perceptible "when the quantity of arsenic was reduced by dilution to one 50,000th of a grain. When farther diluted, the yellowness was gradually less and less discernible, and the precipitate appeared of a light blue. It retained this color until its quantity became too minute for observation. A bluish cloud was, however, very distinctly visible, when the fluid examined contained only the 250,000th part of a grain of arsenic."

Dr. Bostock has presented the society with the detail of some some ingenious experiments on the serum of the blood, from which he deduces the following conclusions:

"1. The serosity of the blood contains no jelly. 2. It contains a minute quantity of albumen. 3. It contains about two per cent. of solid contents; the chief part of which is an animal matter different both from jelly and albumen. 4. It contains a little muriate of soda, and probably also a minute quantity of uncombined alkali. 5. The animal matter peculiar to serosity is not affected either by the oxymuriate of mercury or by tan, and it is not, like albumen, rendered insoluble by heat. 6. The specific gravity of serum is generally not more than 1·023. 7. Its solid contents are generally about twelve per cent. 8. The quantity of uncombined alkali in one ounce of serum is saturated by one grain of sulphuric acid. 9. The alkali is in the caustic state. 10. Serum contains about $\frac{1}{300}$ of its weight of the muriate of soda. 11. It is probable the coagulation of albumen by heat does not depend upon any chemical change in the relation of the alkali to the albumen. 12. It is probable that alcohol coagulates albumen principally by the sudden abstraction of water from it. 13. Sulphuric acid acts partly on the same principle, but partly also from a chemical union between the acid and the albumen. 14. The oxymuriate of mercury acts by forming a chemical compound with the albumen which is insoluble in water. 15. The compound of the oxymuriate of mercury and albumen is not uniform in the proportion of its constituents."

A letter from Mr. Fergusson, inspector-general of hospitals to the army in Portugal, on the mercurial plan of treatment in dysentery, and in yellow and remittent fevers, contains much practical information, which is the more valuable from being the result of considerable experience.

The universal epidemic that prevailed among the military in the field was dysentery. When mild it admitted of an easy cure, by acting on the bowels with mild purgatives, and keeping up their action steadily, but not violently, for a few days. It was also cured, Mr. Fergusson remarks, "with nearly the same facility by acting upon the skin without purgatives, through the influence of active diaphoretics. In this way, every regimental surgeon, looking to the number of recoveries from his sick list, believed that he possessed a cure for the disease, whether he followed the one or the other mode of treatment. The attack, however, sometimes began with such urgent and violent symptoms, that a power beyond either of these two became necessary to save the patient; irreparable mischief otherwise ensued, from the violent inflammation, followed by ulceration and thickening of the colon and larger intestines, and the patient, if he survived the acute attack, sunk afterwards a miserable victim of chronic disease."

In this aggravated form, there appeared to the author one

K 2 nevernever-failing symptom, which always served him as a guide and diagnostic: the urine was high colored, even green, scanty, and pungent; and, though there were no other signs of hepatic affection, it was his signal for beginning and pushing the use of mercurial remedies. "Half a grain of calomel, with one grain of ipecacuan, was given every hour. This never aggravated the abdominal pains; on the contrary, appeared to alleviate them; or, if it did not, some mild saline purgative, as castor-oil largely diluted with mucilage of gum-arabic gave ease, and permitted the continuance of the mercury till the gums were affected. This generally took place in forty-eight hours, when a solution of the disease might be looked for with confidence; and of which one of the most certain precursors was the urine reassuming its natural condition. In a few cases, and really in a very few, the disease did not yield after the mouth became sore, and these were then found to be of an obstinate and incurable nature. I had few opportunities of seeing many of that description, but those that came under my view were elderly soldiers, who, from former abuse of spirituous liquors, might be supposed to labor under previously obstructed viscera. In others, again, the use of mercury was neglected, and the patient died. The dissection then exhibited a miserable mass of disease in the great intestines, the colon, its descending portion particularly, being thickened, knotted, and ulcerated, to an inconceivable degree. The smaller intestines shewed little or nothing of these appearances, and we often lost even the traces of disease, till we came to the liver, which uniformly was blackish, hard, and wasted; the gall-bladder flaccid, and about half full of thin watery bile."

The author found that opium, in almost every stage of the acute disease, was hurtful, and even dangerous; the temporary ease which it afforded from the tormena, being generally followed by worse symptoms. Astringents of every kind, during the acute stage, were even worse than opium.

From all these facts, he concluded that we are to look to the liver principally for the source of the disease. He also contends that dysentery is in no case contagious. It is "the offspring of heat and moisture; of moist cold in any shape after excessive heat: nothing that a man can possibly eat or drink would ever give him true dysentery. A chill from dry cold air would produce a different affection; but the more penetrating forms of moisture, such as lying on damp ground in the hot season, or being exposed to the night dews, or to a stream of chilling exhalations after violent rains, when the system has been relaxed by previous heat, seldom fail to bring it on."

(To be continued.)

*Pharmacologia; or,* The History of Medicinal Substances: *in order to enable the Practitioner to prescribe them with Efficacy and Elegance, and to dispense them with Accuracy. By* JOHN AYRTON PARIS, M.B. F.L.S. 12mo. London, 1812. pp. 229.

"IN an age which is inundated with a profusion of volumes in every department of medical study, a new work must possess more than slender claims to originality, or the publication will be at once blighted by indifference, and its author very justly consigned to reprehension; the discovery of new facts in medical science, can be alone anticipated from those whose sole life is devoted to its research, but the arrangement and application of what is already known, for improving the medical profession, may be reasonably expected from the pen of the practical physician; it is exclusively in the attainment of such an object, that the present work grounds its claims to public notice."

The author of this little volume very fairly, in the above passage in his preface, meets the public opinion. We agree with him that the profuse inundation of volumes professing to teach the art of medicine, is an urging evil of the present day. The legion of vade-mecums and tabular illustrations, emasculated things cut out of better books, certainly call for reprehension; and, if the severity of criticism could correct this abuse, its castigation would be fitly employed on the puny multitude. Dr. Paris, however, will escape the chastisement, from having given an air of originality to his little volume, by inserting, in a more distinct and pointed manner, the chemical incompatibility of compounding certain substances with others in extemporaneous prescription; and by this, as far as he goes, has done a real service to the young physician, and possibly to the veteran practitioner.

In treating his subject, he adopts the subsequent arrangement: in giving the history of each article he notices the sensible qualities; its chemical composition, or the constituents in which its medicinal activity resides; its relative solubility in different menstrua, or the proportions in which it should be combined with different bodies, in order to produce suspension or saturation; the incompatible substances, i. e. all those which are capable of destroying its properties, or rendering its flavor, or aspect, unpleasant or disgusting; the best forms in which it can be exhibited; its specific basis; its medicinal effects; its officinal preparations; and its adulterations.

In justification of the attention he has given to the chemical combinations and their results, as applicable to medicines; and the probable changes that are thus produced

 both both on the form and medicinal properties of substances; he observes,

"The changes, and modifications of which remedies are susceptible, by being submitted to various operations, or mutually combined with each other, are not imaginary, nor are they, as some have supposed, the mere suggestions of theoretical refinement; thus, for instance, every practitioner may easily prove that vegetable tonics lose their astringent character by combinations with alkalies; or, that the efficacy of antimonial preparations is destroyed by vegetable infusions; in the hands, therefore, of criticism and ignorance, valuable remedies may become impotent; and inert medicines converted into poisons. *Unda dabit flammas, et dabit ignis aquas*, may we not to such a cause ascribe the various revolutions which medicinal substances have undergone in the opinions and faith of man; and explain by it the ephemeral reputation of many of those remedies, which, like passing spectres, have glared only for a time, and vanished?"

By copying an article or two we shall present our readers with a specimen of the manner in which the author treats the subject.

"ARSENICI *Oxydum*, Lond. Arsenici, Edin. Arsenicum, Dub. *White Arsenic.*

"QUALITIES. *Form*, shining semivitrious lumps. *Taste*, acrid and corrosive.—CHEMICAL COMPOSITION. Arsenic 75·2, oxygen 24·8; it possesses some of the characteristic properties of an acid, and hence it has been termed *arsenous acid.*—SOLUBILITY. It is very sparingly soluble in water, alcohol, and oil.—FORMS OF EXHIBITION. In pills, by rubbing one grain with ten of sugar, and then heating the mixture with a sufficient quantity of crumb of bread, so as to form ten pills, one of which is a dose; it is, however, most manageable in solution, vid. *Liquor Arsenicalis.**—ADULTERATIONS. It is often sophisticated with chalk, which may be detected by the substance not being entirely volatilized by heat.

"METHOD OF DISCOVERING ITS PRESENCE.—Dissolve one part of the suspected powder, and three parts of subcarbonate of potass, in boiling distilled water, and then slightly touch the surface of the solution with a piece of the nitrate of silver; if any arsenic be

---

* LIQUOR ARSENICALIS. Lond. This is, generally speaking, a solution of the *Arsenite of Potass*; ʒj. contains gr. ſs. of the oxide of arsenic. It was introduced into practice by Dr. Fowler, as a substitute for the empirical remedy, known by the name of "*The Tasteless Ague Drop.*"—INCOMPATIBLE SUBSTANCES. *Lime-water; nitrate of silver; hydrosulphuret of potass; infusions, or decoctions of bark.* Dose, ♏ iv. gradually increasing to ♏ xxx. given twice a-day.

The arrangement of Dr. Paris's book being alphabetical, the Liquor Arsenicalis, which we have inserted in a note, is found under the letter L in his volume.

present, a beautiful yellow precipitate will immediately proceed from the point of contact."

"FERRI SULPHAS. Lond.—Sulphas Ferri. E. D. *Sulphate of iron*, formerly. *Green Vitriol.*

"QUALITIES.—*Form*, crystals, which are rhomboidal prisms, transparent, and of a fine green color, when exposed to the air, they effloresce, and at the same time assume a yellow hue, owing to the attraction of oxygen.—CHEMICAL COMPOSITION. Oxide of iron 28·3, sulphuric acid 26·7, water 45.—SOLUBILITY. ℥j. of water dissolves more than ʒij. of this salt; but it is insoluble in alcohol.—INCOMPATIBLE SUBSTANCES. *All alkaline salts; tartaric acid; borate of soda; nitrate of potass; muriate of ammonia; tartrate of potass and soda; acetate of ammonia; tartrate of potass; muriate of lime; magnesia; and most earthy bodies; nitrate of silver; acetate and superacetate of lead; and almost every salt whose base forms an insoluble compound with sulphuric acid; soaps; sulphurets of potass, and antimony; and astringent vegetables.*—FORMS OF EXHIBITION. It may be administered in solution, or in the form of pill. *Vid. Mist. Ferri.** DOSE, grs. ij. to x. or more."

"DATURA STRAMONIUM, Edin. Stramonium, Dub. Thorn-Apple.

"This plant consists of gum, resin, carbonate of ammonia, and the narcotic principle. Its root smoked in the manner of tobacco, has been lately much extolled as a remedy in the paroxysm of spasmodic asthma; it is, however, a dangerous application, especially in apoplectical habits: the same transient feelings of relief may be procured by smoking a mixture of tobacco and opium."

The utility of this volume is extended by the formula of many nostrums which it contains: and upon the whole we must consider it as an useful manual, and much superior to the common run of vade-mecums and conspectuses.

---

* MISTURA FERRI COMPOSITA L. Compound mixture of iron.

This combination is nearly the same as the celebrated *antihectic mixture* of Dr. Griffith.—CHEMICAL COMPOSITION. It affords a striking example of a new and powerful remedy being produced by the mutual action of the ingredients of a prescription on each other; the new products are *sulphate of potass*, which is dissolved, and *sub-carbonate of iron*, which is diffused, through the mixture, and suspended by the myrrh, which forms a saponaceous compound with the excess of alkali. Its great superiority depends upon the iron being at a *minimum* of oxidation, which renders it more active than the common carbonate, and less irritating than the sulphate; hence its ingredients should be quickly mixed together, and to preserve its virtues should be kept in bottles closely stopped; it is however preferable, that it should be extemporaneously made. Its change of color will generally indicate its loss of efficacy. Dose, ℥j. to ℥ij. twice or thrice a-day.

Report

*Report on the Medicinal Effects of an aluminous Chalybeate Water, lately discovered at Sandrocks, in the Parish of Chale, in the Isle of Wight; pointing out its Efficacy in the Walcheren and other Diseases incident to Soldiers who have served abroad, and more particularly the Advantages to be derived from its Introduction into private Practice.* By William Lempriere, M.D. Physician to the Forces at the Army Depôt. 8vo. pp. 88. Murray.

In this Treatise Dr. Lempriere has undertaken to introduce to our notice an aluminous chalybeate spring, first discovered by Mr. Waterworth, of Newport, in the Isle of Wight. It is situated in the midst of a bold rocky scenery, on the south-west coast, at an elevation of about one hundred feet above the level of the sea. Its temperature was 51°, that of the atmosphere being 48°. From the result of an analysis of the water by Dr. Marcet, it appears that each pint of sixteen ounces contains the following ingredients:

| | Grains. |
|---|---|
| " Of carbonic acid gas, three-tenths of a cubic inch. | |
| " Sulphat of iron in the state of crystallized green sulphat | 41,4 |
| " Sulphat of alumina, a quantity which, if brought to the state of crystallized alum, would amount to . | 31,6 |
| " Sulphat of lime, dried at 160° . . . . . . . . . | 10,1 |
| " Sulphat of magnesia, or Epsom salt, crystallized . . | 3,6 |
| " Sulphat of soda, or Glauber salt, crystallized . . . | 16,0 |
| " Muriat of soda, or common salt, crystallized . . . | 4,0 |
| " Silica . . . . . . . . . . . . . . . . . . . | 0,7 |
| | 107,4 |

From the superior strength of this water, it has been found requisite to commence using it in very small proportions. The cases in which it has proved efficacious, are such as are usually benefited by chalybeates. Dr. Lempriere, indeed, had an opportunity of giving it in some of those deplorable cases of debility which occurred after the severe diseases to which so many of our soldiers were subjected at Walcheren. The beneficial effect of the water was very speedily evident in the improvement which took place in the appetite and spirits of the exhausted and worn-down patients; and the recovery proved more permanent than in those cases in which other remedies had been employed.

---

*Narrative respecting the Case of confirmed Cancer, which was speedily and radically cured, by an external Application only.* 8vo. Murray. pp. 23.

Next to the duty of directing our readers to where they may gain information on medical subjects, and the quality of of that information is that of pointing out to them where no medical information is to be had, notwithstanding the alluring position of a title-page. This narrative contains no intimation of the name of the material employed thus successfully, as it is said. Every thing that could lead others to a trial of it is carefully concealed; but the residence and name of the doctor who cures confirmed cancers in a fortnight, is given with minute precision.

